# *Notes from the Field:* Increases in Imported Malaria Cases — Three Southern U.S. Border Jurisdictions, 2023

**DOI:** 10.15585/mmwr.mm7318a2

**Published:** 2024-05-09

**Authors:** Cedar L. Mitchell, Audrey Kennar, Yvonne Vasquez, Kaitlyn Noris, Thomas Williamson, Andrea Mannell, Anissa Taylor, Irene Ruberto, Theresa A. Cullen, Mariana Singletary, Seema Shah, Hector Ocaranza, Alfonso Rodriguez Lainz, Kimberly E. Mace

**Affiliations:** ^1^Epidemic Intelligence Service, CDC; ^2^Pima County Health Department, Tucson, Arizona; ^3^Arizona Department of Health Services; ^4^Division of Global Migration Health, National Center for Emerging and Zoonotic Infectious Diseases, CDC; ^5^County of San Diego Health and Human Services Agency, San Diego, California; ^6^City of El Paso Department of Public Health, El Paso, Texas; ^7^Division of Parasitic Diseases and Malaria, National Center for Emerging and Zoonotic Infectious Diseases, CDC.

**Summary**

**What is already known about this topic?**

Approximately 2,000 malaria cases are imported into the United States annually, mostly among U.S. residents with recent travel to areas with endemic malaria.

**What is added by this report?**

In 2023, reports of imported malaria in three U.S. southern border jurisdictions increased from cases reported in 2022. Enhanced case investigations documenting traveler residency indicated higher percentages of malaria infections among new arrivals to the United States who traveled through at least one country with endemic malaria, including crossing land borders.

**What are the implications for public health practice?**

Outreach and education about malaria should be provided to local health care professionals and new arrivals, including migrants, with travel through areas with endemic malaria, to facilitate identification of cases, initiation of prompt treatment, and reduction in morbidity.

## Introduction

Malaria is a severe and potentially fatal mosquitoborne disease caused by infection with *Plasmodium *spp. parasites. Although malaria is no longer endemic in the United States, imported infections are reported annually; the primary risk group has been U.S. residents traveling to areas where malaria is endemic (*1*). In 2023, sporadic locally acquired mosquito-transmitted malaria cases were reported in several U.S. states (*2*,*3*). This report describes increases in imported malaria cases in 2023 compared with 2022 in three public health jurisdictions along the U.S. southern border.

## Investigation and Outcomes

During January–December 2023, a total of 68 imported malaria cases were identified from reportable disease surveillance systems in Pima, Arizona (18), San Diego, California (27), and El Paso, Texas (23), compared with 28 cases in 2022 (three in Pima, 12 in San Diego, and 13 in El Paso) (Table). Because malaria case counts were higher than expected, enhanced case investigations were initiated. Malaria cases were defined according to CDC case definitions.* To describe imported malaria cases in these three jurisdictions, this report summarized patient travel and illness characteristics by U.S. residence status. New arrivals were non–U.S.-born persons who had arrived in the United States within the preceding 6 months and were classified into the following three subgroups: 1) newly arrived refugees (i.e., officially admitted to the United States as part of the U.S. Refugee Admissions Program), 2) other new arrivals (including asylum seekers and other migrants), and 3) persons whose immigration status was unknown. Among jurisdictions, differences were identified in epidemiologic investigation protocols for patients without a local address and whether they were included in local surveillance case counts. This activity was reviewed by CDC, deemed not research, and was conducted consistent with applicable federal law and CDC policy.^†^

**TABLE:** Characteristics of imported malaria cases reported among patients, by U.S. residency and new arrival visa status —three southern U.S. border jurisdictions,*^,^^†^ 2023

Characteristic	No. (row %)				
	Total	U.S. residents	Newly arrived refugees^§^	Other new arrivals^¶^	New arrivals with unknown immigration status**
**Total **	**68**	**15 (22)**	**2 (3)**	**49 (72)**	**2 (3)**
**Surveillance and data collection**					
Patient interview attempted	**61 (90)**	15 (100)	2 (100)	42 (86)	2 (100)
Case investigation completed^††^	**61 (90)**	14 (93)	2 (100)	44 (90)	1 (50)
Case included in local surveillance data**^§§^**	**33 (49)**	15 (100)	2 (100)	16 (33)	0 (—)
**Travel type**					
Land border crossing only	**26 (38)**	0 (—)	0 (—)	26 (53)	0 (—)
Air travel only	**20 (29)**	15 (100)	2 (100)	2 (4)	1 (50)
Air travel and land border crossing	**10 (15)**	0 (—)	0 (—)	10 (20)	0 (—)
Unknown	**12 (18)**	0 (—)	0 (—)	11 (22)	1 (50)
**Complexity of travel**					
Direct travel to U.S. destination from country with endemic malaria	**18 (27)**	15 (100)	2 (100)	0 (—)	1 (50)
Transit through one or more country with endemic malaria	**46 (68)**	0 (—)	0 (—)	46 (94)	0 (—)
Unknown travel	**4 (6)**	0 (—)	0 (—)	3 (6)	1 (50)
**Region of travel origin^¶¶^**					
Africa	**29 (43)**	14 (93)	2 (100)	12 (25)	1 (50)
Asia	**9 (13)**	1 (7)	0 (—)	8 (16)	0 (—)
Central America	**3 (4)**	0 (—)	0 (—)	2 (4)	1 (50)
South America	**27 (40)**	0 (—)	0 (—)	27 (55)	0 (—)
**No. of regions traveled through**					
1	**19 (28)**	14 (93)	2 (100)	2 (4)	1 (50)
≥2	**47 (69)**	1 (7)	0 (—)	46 (94)	0 (—)
Unknown	**2 (3)**	0 (—)	0 (—)	1 (2)	1 (50)
Days from symptom onset to seeking care,*** median (IQR)	**6 (4–11)**	6 (4–7)	18 (10–25)	6 (4–13)	1 (1–1)
Days from symptom onset to diagnosis,*** median (IQR)	**7 (4–13)**	7 (5–11)	21 (15–26)	7 (4–14)	7 (7–7)
**Malaria species reported**					
*Plasmodium vivax*	**34 (50)**	2 (13)	0 (—)	31 (63)	1 (50)
*Plasmodium falciparum*	**21 (31)**	10 (67)	0 (—)	11 (22)	0 (—)
*Plasmodium malariae*	**4 (6)**	0 (—)	2 (100)	1 (2)	1 (50)
*Plasmodium ovale*	**1 (2)**	0 (—)	0 (—)	1 (2)	0 (—)
Undetermined	**8 (12)**	3 (20)	0 (—)	5 (10)	0 (—)
**Hospitalization**					
Hospitalized	**62 (91)**	12 (80)	1 (50)	47 (96)	2 (100)
Not hospitalized	**5 (7)**	3 (20)	1 (50)	1 (2)	0 (—)
Unknown	**1 (2)**	0 (—)	0 (—)	1 (2)	0 (—)
**Disease severity**					
Severe malaria^†††^	**21 (31)**	1 (7)	0 (—)	18 (37)	2 (100)
Severity unknown	**10 (15)**	3 (20)	0 (—)	7 (14)	0 (—)
Death	**0 (—)**	0 (—)	0 (—)	0 (—)	0 (—)
**Available for follow-up after treatment**	**18 (27)**	10 (67)	1 (50)	7 (14)	0 (—)

* Jurisdictions included Pima, Arizona; San Diego, California; and El Paso, Texas.

^† ^During 2022, a total of 28 imported malaria cases were reported from these three jurisdictions, including 15 (54%) among U.S. residents, zero among newly arrived refugees, 11 (39%) among other new arrivals, and two (7%) among persons with an unknown immigration status.

^§^ Refugees from areas in sub-Saharan Africa with endemic malaria receive presumptive treatment for malaria during their predeparture health assessment. https://www.cdc.gov/immigrantrefugeehealth/guidelines/overseas-guidelines.html

^¶ ^Asylum seekers and other migrants.

** Includes one short-term traveler to the United States and one patient without enough information to determine their status.

^†† ^Case investigation protocols differed among jurisdictions. Some protocols required interviews for all reported patients, whereas others only required interviews for patients with a local residential address. Reasons for an incomplete case investigation included inability to contact the patient, and loss to follow-up because of missing or incorrect patient contact information or no response.

^§§ ^Inclusion criteria for local surveillance counts differed among jurisdictions. Some jurisdictions did not include patients who were missing a residential address or whose address was outside the local jurisdiction, regardless of case investigation status.

^¶¶ ^Region of travel origin for new arrivals or region of destination for U.S. residents. Regions included the following countries of travel origin: *Africa:* Angola, Côte d’Ivoire, Ethiopia, Guinea, Mauritania, Nigeria, Senegal, Sudan, The Gambia, and Uganda; *Asia: *Afghanistan and China; *Central America:* Nicaragua and Panama; *South America:* Colombia, Ecuador, and Venezuela. CDC provides information about areas with endemic malaria. https://wwwnc.cdc.gov/travel/yellowbook/2024/infections-diseases/malaria

*** Date of care and diagnosis based on care received at a U.S. health care facility.

^††† ^According to the CDC case definition for severe malaria, which includes laboratory confirmation with neurologic symptoms, acute kidney injury, severe anemia (hemoglobin <7g/dL), acute respiratory distress syndrome, or ≥5% parasitemia; treatment for severe malaria (i.e., artesunate or exchange transfusion); or death. https://doi.org/10.15585/mmwr.ss7108a1

Among the 68 imported malaria cases identified in 2023, 15 (22%) occurred among U.S. residents, two (3%) among newly arrived refugees, 49 (72%) among other newly arrived migrants (i.e., asylum seekers and other migrants), and two (3%) among travelers with unknown immigration status. The local public health jurisdictions attempted an interview with 61 (90%) patients. Among the 68 patients with malaria, 33 (49%) met residence criteria for inclusion in local surveillance case counts (i.e., the 15 U.S. residents, two newly arrived refugees, and 16 [33%] of the 49 other newly arrived migrants). The U.S. residents and refugees traveled directly from another country with endemic malaria to the United States. Among the 49 other newly arrived migrants, 46 (94%) had traveled through one or more countries with endemic malaria, including the country of origin (complex travel). The median travel duration was 29 days (range = 8–85 days), and 36 (73%) persons reported having traversed land borders. Overall, 63 (91%) patients with malaria were hospitalized; no deaths were reported. Nearly one third (21; 31%) of patients with malaria experienced severe disease (*1*), of which *Plasmodium vivax* was reported among 11 (52%), *P. falciparum* among six (29%), and another or unknown *Plasmodium *spp. parasite among four patients. Severe malaria was more common among other newly arrived migrants (18 of 49; 37%) than among U.S. residents (one of 15; 7%).

## Preliminary Conclusions and Actions

Imported malaria in three U.S. southern border jurisdictions increased in 2023, particularly among new arrivals to the United States with recent, complex transit through at least one country with endemic malaria. During the same period, entry of asylum seekers and other migrants into the United States across the southern land border increased.^§^ In light of the different jurisdictional protocols used in case investigations, implementation of classifications and consistent investigation and reporting protocols for non-U.S. residents could facilitate better characterization of malaria incidence among new arrival subgroups in different jurisdictions.^¶^

New arrivals to the United States with complex travel through areas with endemic malaria are potentially at higher risk for malaria and, for reasons not fully understood, for more severe illness. Health care professionals should obtain a complete travel history, consider malaria among symptomatic patients with recent travel through areas where malaria is endemic, and initiate prompt testing and, if indicated, treatment.** Outreach and education about malaria directed to local health care professionals and to new arrivals with recent travel in areas with endemic malaria are crucial because prompt care seeking, diagnosis, and treatment of malaria will reduce morbidity in this population.

## Footnotes

* https://ndc.services.cdc.gov/case-definitions/malaria-2014/^†^ 45 C.F.R. part 46, 21 C.F.R. part 56; 42 U.S.C. Sect. 241(d); 5 U.S.C. Sect. 552a; 44 U.S.C. Sect. 3501 et seq.

§ An increase in the entry of asylum seekers and other migrants across the U.S. southern border was identified using annual numbers of persons with credible fear who were released with a notice to appear for immigration court or paroled into the United States as a proxy for asylum seekers and other migrants. Data are publicly available from 2022 and 2023 annual U.S. Customs and Border Protection Southwest border reports. https://www.cbp.gov/newsroom/stats/custody-and-transfer-statistics

¶
https://cdn.ymaws.com/www.cste.org/resource/resmgr/PS/03-ID-10revised.pdf

**
https://www.cdc.gov/malaria/diagnosis_treatment/clinicians1.html
